# Differences in Plantar Flexor Fascicle Length and Pennation Angle between Healthy and Poststroke Individuals and Implications for Poststroke Plantar Flexor Force Contributions

**DOI:** 10.1155/2014/919486

**Published:** 2014-07-23

**Authors:** John W. Ramsay, Thomas S. Buchanan, Jill S. Higginson

**Affiliations:** ^1^Biomechanics and Movement Science Program, University of Delaware, Newark, DE 19716, USA; ^2^Delaware Rehabilitation Institute, University of Delaware, Newark, DE 19713, USA

## Abstract

Poststroke plantar flexor muscle weakness has been attributed to muscle atrophy and impaired activation, which cannot collectively explain the limitations in force-generating capability of the entire muscle group. It is of interest whether changes in poststroke plantar flexor muscle fascicle length and pennation angle influence the individual force-generating capability and whether plantar flexor weakness is due to uniform changes in individual muscle force contributions. Fascicle lengths and pennation angles for the soleus, medial, and lateral gastrocnemius were measured using ultrasound and compared between ten hemiparetic poststroke subjects and ten healthy controls. Physiological cross-sectional areas and force contributions to poststroke plantar flexor torque were estimated for each muscle. No statistical differences were observed for any muscle fascicle lengths or for the lateral gastrocnemius and soleus pennation angles between paretic, nonparetic, and healthy limbs. There was a significant decrease (*P* < 0.05) in the paretic medial gastrocnemius pennation angle compared to both nonparetic and healthy limbs. Physiological cross-sectional areas and force contributions were smaller on the paretic side. Additionally, bilateral muscle contributions to plantar flexor torque remained the same. While the architecture of each individual plantar flexor muscle is affected differently after stroke, the relative contribution of each muscle remains the same.

## 1. Introduction

Stroke is a leading cause of long-term adult disability in the United States. It has been reported that approximately 795,000 American adults are affected by a stroke each year and that the prevalence of stroke will increase by an estimated 25% by 2030 [1]. Muscle weakness contralateral to the brain lesion, or hemiparesis, is the most common impairment following stroke [2, 3] and is evident by a decrease in maximal voluntary strength on the paretic limb compared to the nonparetic limb [4, 5].

A combination of muscular and neurological impairments is believed to contribute to poststroke hemiparesis [6]. Since the force-generating capacity of a muscle is dependent on amount of impairment, some recent studies have identified the extent to which these changes occur after stroke. Using magnetic resonance imaging, Ramsay et al. [7] observed muscle atrophy in twelve out of fifteen lower extremity muscles. They found an overall decrease in contractile tissue of 20% in the shank area and 24% in the thigh. Similarly, Klein et al. [8] also observed muscle atrophy in the plantar flexor muscles but additionally found that plantar flexor weakness arises primarily from muscle activation failure. Paretic plantar flexor muscle activation was impaired by 50% or more when compared to the nonparetic side. Knarr et al. [9] reported that plantar flexor muscle volumes derived from MRIactually underestimate the deficit in maximum force generating ability by approximately 15%. Therefore, while muscle atrophy and impaired activation have been reported, additional changes in the structure of poststroke muscles likely exist and may be necessary to measure when attempting to accurately describe the force-generating potential of individual poststroke muscles.

Structural changes that occur at the muscle fascicle level may include lengthening or shortening of the fascicle or a change in the orientation (i.e., pennation angle) of the muscle fibers as they span the muscle belly. Ultrasonography is a cost-effective and noninvasive method for measuring the structure of an individual muscle* in vivo*. Using ultrasound, Gao et al. [10] have shown that medial gastrocnemius fiber lengths and pennation angles are smaller after stroke compared to healthy controls, which has implications for changes in both active and passive muscle properties. However, little is known about other plantar flexor muscles (e.g., lateral gastrocnemius, soleus) or to what extent changes occur in the nonparetic leg. Additionally, understanding how the changes in muscle properties affect the percentage of force contribution of each plantar flexor muscle to overall plantar flexor torque will give insight into the functional use of each muscle after stroke and whether poststroke plantar flexor weakness is due to uniform changes in individual muscle forces.

The objective of this study was twofold; first, we aimed to quantify poststroke muscle fascicle lengths and pennation angles of three individual plantar flexor muscles (i.e., medial and lateral gastrocnemius, soleus) and compare them to healthy controls. We hypothesized that in chronic stroke survivors, plantar flexor muscle fascicle lengths and pennation angles would not change between paretic and nonparetic sides but would be smaller compared to healthy controls. Secondly, we aimed to estimate the percentage of force contribution of each muscle to overall plantar flexor torque for each of our poststroke subjects.

## 2. Methods

### 2.1. Subjects

Ten poststroke subjects (61 ± 10 yrs, 8 males, 52 ± 40 months since stroke) and ten healthy control subjects (54 ± 11 yrs, 6 M) were included in this study. A complete summary of subject demographics can be found in Table 1. Inclusion criteria were (1) chronic stroke occurring at least six months previously; (2) single lesion; (3) age 30–80 years; and (4) ambulatory but with noticeable gait deficits. Subjects were excluded if they had (1) multiple strokes affecting both sides of the body; (2) heart disease or hypertension; (3) dementia; (4) severe aphasia; (5) orthopedic or pain conditions; (6) cancer; (7) any metal implants; or (8) claustrophobia. All subjects signed an informed consent approved by the University of Delaware review board.

### 2.2. Muscle Architecture (*L* ^*f*^, *θ*)

Each subject was seated in a Biodex dynamometer (Biodex Medical Systems, Shirley, New York) with their knee fully extended and their foot secured at neutral ankle flexion in a foot plate. Velcro straps were used to prevent the foot from moving. An additional restraint was used to prevent the knee from bending. All measurements were collected during a resting condition.

**Figure 1:**
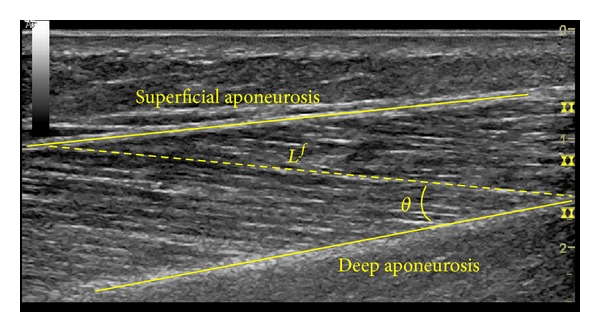
Longitudinal ultrasound image of the lateral gastrocnemius muscle. Top solid yellow line marks the superficial aponeurosis and the deep solid yellow line marks the deep aponeurosis. *L* ^*f*^ follows the fascicle length from deep to superficial aponeuroses, and *θ* is the angle between Lf and deep aponeurosis.

Individual muscle fascicle lengths (*L* ^*f*^) and pennation angles (*θ*) were measured using a GE LOGIQ P6 (GE Healthcare, Waukesha, WI, USA) ultrasound device. Three ankle muscles were measured: the medial gastrocnemius (MG), lateral gastrocnemius (LG), and soleus (SOL). Longitudinal ultrasound images were recorded using a B-mode scanner with a 15 MHz high-resolution linear array probe (ML6-15). Scanning parameters were optimized to obtain the highest quality of ultrasound image for each subject. To account for the possibility of nonuniform fascicle lengths along the length of the muscle, *L* ^*f*^ and *θ* were recorded at the midbelly of each muscle. *L* ^*f*^ was measured between deep and superficial aponeuroses and *θ* was measured as the angle between the deep aponeurosis and the muscle fascicle itself (Figure 1). When the full length of a muscle fascicle was outside the field-of-view of the linear probe, a technique of extended field-of-view called LOGIQ View was used. LOGIQ View has been shown to be accurate within 5% [11]. Two measurements were taken for *L* ^*f*^ and *θ* and an average between the two was calculated as a representative value for that parameter. For stroke subjects, the paretic and nonparetic limbs were treated separately, whereas, for the control subjects, an average of both sides was used as a general comparison between the two poststroke groups.

### 2.3. Plantar Flexor Force Contributions

Physiological cross-sectional area at neutral position (PCSA) for each poststroke plantar flexor muscle was estimated using muscle volume reconstruction techniques from previous work [7]. For all subjects, axial T1-weighted MR images were acquired for both legs using a 1.5 T Signa LX scanner (GE Medical, Milwaukee, WI). IMOD software (University of Colorado, Boulder, CO; [12]) was used to manually trace the boundary of each individual plantar flexor muscle over the entire muscle length. Cross-sectional areas were adjusted for noncontractile tissue using a pixel threshold and volume was calculated by summing the adjusted cross-sectional areas and multiplying by the slice thickness (11.5 mm) over the length of the muscle belly. Muscle volumes for six of the ten poststroke subjects (subjects 1–6) in the current study were included in the analysis performed by Ramsay et al. [7].

PCSA is a function of the muscle volume, fascicle length, and pennation angle and was estimated using

(1) $$\begin{array}{c} \text{PCSA}=\frac{V^{m}}{L^{f}}\cos\left(\theta \right), \end{array}$$

where *V* ^*m*^ is the muscle volume at neutral position. To determine the relative contribution, *F*_*m*_, of each muscle to the combined plantar flexor joint torque (*τ* ^max⁡^), the ratios of individual muscle PCSA were determined. This required the assumptions that muscle stress remains the same after stroke or that any change in poststroke muscle stress is uniform across the muscle group and that PCSA changes linearly with increasing force. Under these two assumptions,

(2) $$\begin{array}{c} \sigma =\frac{F_{\text{MG}}}{{\text{PCSA}}_{\text{MG}}}=\frac{F_{\text{LG}}}{{\text{PCSA}}_{\text{LG}}}=\frac{F_{\text{SOL}}}{{\text{PCSA}}_{\text{SOL}}}. \end{array}$$

Rearrange (2) to obtain PCSA ratios:

(3) $$\begin{array}{c} \frac{F_{\text{MG}}}{F_{\text{LG}}}=\frac{{\text{PCSA}}_{\text{MG}}}{{\text{PCSA}}_{\text{LG}}}=P;\;\;\frac{F_{\text{LG}}}{F_{\text{SOL}}}=\frac{{\text{PCSA}}_{\text{LG}}}{{\text{PCSA}}_{\text{SOL}}}=Q. \end{array}$$

And thus, *F*_MG_ = *PF*_LG_ and *F*_LG_ = *QF*_SOL_.

Finally, using true (i.e., not volitional) *τ* ^max⁡^ obtained from burst superimposition methods [9], we determined the relative contribution of each individual muscle to plantar flexion torque:

(4) τmax⁡=FMG·rMG+FLG·rLG+FSOL·rSOL=FSOL[(P·Q·rMG)+(Q·rMG)+rSOL]=FSOL[R],

which can be rearranged so that *F*_SOL_ = *τ* ^max⁡^/*R*, where *r* is the moment arm of each muscle at neutral joint angle and *R* is a constant derived from all known values. Moment arms were taken from a subject-specific musculoskeletal model in OpenSim [13] that had been geometrically scaled to each individual subject. Multiplying each *F*_*m*_ by its respective moment arm and dividing by *τ* ^max⁡^, the percent contribution to overall joint torque was calculated.

### 2.4. Statistical Analysis

Fascicle length and pennation angle data were tested for normality using the Lilliefors test, and parametric statistics were used. One-way ANOVAs were used to compare between paretic, nonparetic, and healthy control values for each muscle parameter. Tukey's least significant difference was used to test post-hoc differences between each individual group. A significance level of *α* = 0.05 was used for all statistical tests. PCSA and *F*_*m*_ were compared using the ratio of paretic to nonparetic limb. The percentage of each muscle contribution to overall torque (*τ* ^max⁡^) was also calculated.

## 3. Results

### 3.1. Muscle Architecture

Due to patient time constraints during the data collection, physical fatigue, and muscle cramps, only 2 muscles (MG, SOL) were collected from one poststroke subject (S1; LG *n* = 9). All group means can be found in Table 2. No statistical differences were observed between paretic, nonparetic, and healthy fascicle lengths; however, mean values were smaller for poststroke gastrocnemius when compared to healthy controls by 3–7 mm. There were no significant differences in pennation angle for the LG and SOL between paretic, nonparetic, and healthy limbs. For the MG, there was a significant decrease (*P* = 0.0181) in the paretic limb compared to both nonparetic and healthy limbs.

### 3.2. Plantar Flexor Force Contributions

Mean PCSA and *F*_*m*_ values can be found in Table 3. Two subjects were excluded from force contribution estimations (see Table 1); therefore only 8 subjects were used. All paretic PCSAs were smaller on the paretic side, as indicated by paretic/nonparetic ratios less than 1. Relative force contributions (*F*_*m*_) followed the same trend, as the paretic values were all smaller than the nonparetic side. However, the *F*_*m*_ ratios were much lower (0.66–0.68) for each muscle. As a percentage of total plantar flexor torque, each individual muscle was the same between paretic and nonparetic limbs.

## 4. Discussion

Changes in the force-generating properties and motor control of poststroke muscles can alter the way muscles are able to generate functional movements, which may lead to deviations from healthy patterns. Muscle architectural parameters such as fascicle length and pennation angle are two factors that affect how poststroke muscles contract and generate force. In this study we used medical imaging techniques to measure fascicle length and pennation angle of the poststroke plantar flexors. This data was used to estimate physiological cross-sectional areas and force contributions for each muscle.

In general, our paretic MG fascicle length results and pennation angles and healthy pennation angles were similar to each other and to the values reported previously at neutral ankle angle [10]. While our healthy fascicle lengths were within the range observed by Gao et al. [10], we also found that our healthy values were slightly, but not significantly larger than our paretic values. The discrepancy in the results could be due to the sample used or due to the location along the muscle belly where fascicle length was measured. The previous study used a 5 cm distance from the musculotendon junction for all measurements, where as we took our measurements at the muscle midbelly. Additionally, the previous study recruited subjects with muscle spasticity, while we did not control that.

While no data has been published on poststroke LG or SOL muscles to compare our results with, we believe that the general consistency between MG data strengthens the methodology and results from both current and previous studies. The paretic and nonparetic LG fascicle lengths had lower means than healthy by approximately 7 mm, which was greater than the difference in MG fascicle lengths, but still not significant. Muscle force is transmitted to the tendon along the muscle fascicle axis and is proportional to the cosine of the pennation angle [14, 15]. In the present study, the paretic MG pennation angle was significantly smaller by approximately 5°; however, it is functionally meaningless because the cosine of 5° is so small (~0.003%). Nonetheless, the smaller pennation value does confirm muscle atrophy. By including both medial and lateral heads of the gastrocnemius in our study, we observed that both heads of the gastrocnemius are affected differently after stroke. It seems that the paretic gastrocnemius has a complex mechanism for atrophy involving changes in muscle volume as well as fascicular length and orientation. In addition, we did not investigate changes in individual muscle fiber types, which could also explain the differential effect we observed in the gastrocnemius. Although both Type I and Type II fibers undergo changes in various muscle types [16], previous studies have shown selective Type II fiber atrophy with hypertrophy of Type I muscle fibers [16–18]. Specifically, Dattola et al. [17] found this to be true in the gastrocnemius. This change in fiber type ratio may be indicative of activation failure or disuse specific to the gastrocnemius.

For the SOL we found no differences between poststroke and healthy fascicle length or pennation angle. A previous study did find significantly smaller SOL muscle volumes on the paretic side compared to the nonparetic [7]. Smaller volumes with no change in fascicle length or pennation angle could be due to a decrease in overall muscle length or an increase in number of sarcomeres in series. Gao and Zhang [19] suggested that the shift and increased slope of the poststroke gastrocnemius active force-length relationship may be due to a reduced number of sarcomeres in series along the fascicle. In the present study, no change in the fascicle length between healthy and poststroke SOL suggests either that the opposite is true (i.e., an increase in number of sarcomeres in series) for this muscle or that there is an increase in fascicular tension. Since the paretic Achilles tendon length appears to increase in length after stroke [20], an increase in plantar flexor fascicular tension may be a viable mechanism for the observed changes in muscle architecture. However, further research about the active components of the SOL is warranted to confirm these arguments.

Muscle volume may underestimate the amount of force deficit between paretic and nonparetic limbs and, in addition to impaired activation, these differences may also be a result of changes in the muscle architecture [9]. PCSA incorporates architectural measurements (i.e., *L* ^*f*^ and *θ*) and muscle volume. Our PCSA values reflected plantar flexor volume data from previous studies [8, 9] in that the paretic values were approximately 15% smaller than the nonparetic side. Therefore, these architectural changes may not address the underlying mechanism of poststroke muscle weakness. However, when using the ratios of PCSA to derive muscle force, we found a 66–68% difference in individual muscle force contributions—similar to the force deficits reported in [9]. These estimates were based on similar methodology and ultimately are dependent on values for *τ* ^max⁡^. Interestingly, while force contributions decreased consistently across each plantar flexor muscle, the overall contribution of each muscle to poststroke plantar flexor torque remained the same. That is, regardless of hemiparesis or the actual value for *F*_*m*_, the MG, LG, and SOL remained approximately 23%, 18%, and 59% of *τ* ^max⁡^, respectively. This information may be useful when partitioning unknown forces from a known torque is desired, such as in computational modeling.

We assumed that PCSA, derived from parameters measured in a neutral resting condition rather than at optimal fiber length, is directly proportional to muscle force. Optimal fiber length for the paretic MG decreases by approximately 1.5 cm from healthy [19] which would likely increase the paretic PCSA for this muscle. Since the shift in optimal fiber lengths for the remaining plantar flexors and how these changes compare between paretic and nonparetic muscles is unknown, it is difficult to assess the overall effect that differences in optimal fiber length would have on our current results. However, if the changes remain uniform for all plantar flexors and across limbs, the overall contribution of forces would be unchanged.

Two potential sources of measurement error in our ultrasound measurement technique should be acknowledged. First, in an attempt to minimize the potential effect of fascicle length nonuniformity within a muscle, we measured fascicle lengths and pennation angles at the midbelly of each muscle. While care was taken to approximate the location of the midbelly in a similar manner for all subjects and for all measurements, this is less standardized than imaging from a discrete physiological landmark such as the musculotendon junction. Error may also have been due to the extended field-of-view technique we used which can introduce errors within 5% [11]. These potential measurement errors may explain why our data was not significantly different.

There are a few limitations in the present study. First, we assumed that muscle stress for each individual poststroke plantar flexor was affected similarly. As discussed previously, an increase in fascicular tension may be occurring and may be indicative of changes in stress, but to what degree is currently unknown. By measuring the architectural parameters at neutral joint angle and during a resting condition, we acknowledge that the true contractile properties of each muscle may be different than as reported. As muscle contraction increases, fascicle length will shorten and pennation angle will increase; however, the extent to which the changes in poststroke plantar flexor muscle architecture at different levels of muscle contraction exist and whether the changes are uniform across muscles would be worth investigating. As pennation angle will increase with increased contraction, the contribution to overall muscle force may also be underestimated.

It should also be noted that the stretch reflex initiating a spastic response could have emerged with the knee fully extended during testing as the gastrocnemius is in a stretched position. While most of our subjects had minimal or no presence of clonus, and therefore likely did not have spasticity, we acknowledge that we did not account for this during the ultrasound protocol. We also studied each muscle as a uniarticular muscle, whereas the gastrocnemius is biarticular. To fully understand the changes occurring across the gastrocnemius, evaluating all parameters at varying knee joint angles may provide a more comprehensive understanding of poststroke architectural differences.

## 5. Conclusions

From multiple imaging modalities (i.e., MRI and ultrasound), we have derived architectural parameters for three poststroke plantar flexor muscles and showed subtle differences between paretic, nonparetic, and healthy control limbs. Using these* in vivo *parameters, we subsequently estimated the PCSA and relative force contributions for each poststroke plantar flexor muscle, although these changes do not fully explain altered poststroke force generating capacity. Our results indicate that the architecture of the medial and lateral heads of the gastrocnemius is affected by more than SOL following stroke. We have also shown that, despite the architectural changes between these muscles, the overall relative force contribution of each plantar flexor muscle remains the same regardless of limb.

## Figures and Tables

**Table 1:** Demographics for 10 post-stroke and 10 healthy adults.

Stroke Subject #	Gender	Side of Paresis	Age	Months Since Stroke	Height (m)	Fugl-Meyer Lower Extremity Score	Presence of Clonus	Healthy Subject #	Gender	Age	Height (m)
1∗	M	R	65	89	1.73	23	Sustained	1	M	31	1.83
2	M	R	76	80	1.87	12	None	2	M	63	1.77
3	M	R	62	12	1.74	13	Present^‡^	3	M	60	1.70
4	M	R	51	9	1.80	15	Present^†^	4	F	55	1.66
5	F	L	74	10	1.63	19	None	5	F	44	1.63
6	M	L	59	85	1.80	26	Present^‡^	6	M	54	1.71
7	M	R	63	12	1.80	25	None	7	F	51	1.69
8	M	L	46	23	1.74	23	None	8	M	49	1.93
9	F	R	48	105	1.70	16	None	9	F	74	
10	M	L	69	99	1.78	22	None	10	M	59	1.83

Averages			61	52						54	

*Only two muscles (MG, SOL) were collected due to patient time constraints, physical fatigue and muscle cramps; therefore (*F*_*m*_) was unable to be estimated.

**†**Subject did not have plantar flexor torque testing performed due to clonus during burst testing.

^‡^Clonus was present, but minimal.

**Table 2:** Fascicle lengths and pennation angles for post-stroke and healthy adults (±SD).

	*L*_MG_^*f*^ (m)	*L*_LG_^*f*^ (m)	*L*_SOL_^*f*^ (m)	*θ*_MG_ (°)	*θ*_LG_ (°)	*θ*_SOL_ (°)
Paretic	0.0510 ± 0.0079	0.0549 ± 0.0184	0.0397 ± 0.0104	15.7 ± 3.0	13.1 ± 4.7	18.0 ± 34
Non-Paretic	0.0513 ± 0.0082	0.0545 ± 0.0172	0.0384 ± 0.0106	20.6∗ ± 4.6	14.6 ± 5.3	17.9 ± 5.8
Healthy	0.0547 ± 0.0079	0.0619 ± 0.0075	0.0394 ± 0.0098	19.4∗ ± 2.9	13.7 ± 2.1	18.4 ± 3.8
ANOVA *P*-value	0.5596	0.0868	0.9641	0.0181	0.5079	0.9239

*Significant increase compared to paretic limb.

**Table 3:** PCSA, force contributions (*F*_*m*_) and percentages of overall plantar flexor torque (±SD).

	PCSA_MG_ (cm^2^)	PCSA_LG_ (cm^2^)	PCSA_SOL_ (cm^2^)	*F*_MG_ (N)	*F*_LG_ (N)	*F*_SOL_ (N)	%_MG_^*PF*^	%_LG_^*PF*^	%_SOL_^*PF*^
Paretic	26.22 ± 2.97	20.89 ± 3.24	72.43 ± 26.61	424.30 ± 148.03	330.24 ± 96.46	1099.79 ± 428.73	23.33 ± 6.13	18.4 ± 2.73	58.1 ± 8.56
Non-Paretic	31.27 ± 6.61	24.36 ± 6.89	86.56 ± 27.63	626.71 ± 168.55	483.35 ± 140.92	1676.29 ± 436.94	22.8 ± 3.62	17.83 ± 2.49	59.36 ± 5.41
P/NP ratio	0.84	0.86	0.84	0.68	0.68	0.66	—	—	—
